# Risk stratification and role for additional diagnostic testing in patients with acute chest pain and normal high-sensitivity cardiac troponin levels

**DOI:** 10.1371/journal.pone.0203506

**Published:** 2018-09-07

**Authors:** Martijn W. Smulders, Sebastiaan C. A. M. Bekkers, Yvonne J. M. van Cauteren, Anna Liefhebber, Jasper R. Vermeer, Juliette Vervuurt, Marja P. van Dieijen-Visser, Alma M. A. Mingels, Hans-Peter Brunner-La Rocca, Pieter C. Dagnelie, Joachim E. Wildberger, Harry J. G. M. Crijns, Bas L. J. H. Kietselaer

**Affiliations:** 1 Department of Cardiology, Maastricht University Medical Center, Maastricht, The Netherlands; 2 Cardiovascular Research Institute Maastricht (CARIM), Maastricht University, Maastricht, The Netherlands; 3 Department of Radiology, Maastricht University Medical Center, Maastricht, The Netherlands; 4 Department of Clinical Chemistry, Maastricht University Medical Center, Maastricht, The Netherlands; 5 Department of Epidemiology, Maastricht University, Maastricht, The Netherlands; 6 School for Public Health and Primary Care (CAPHRI), Maastricht University, Maastricht, The Netherlands; 7 Department of Cardiology, Zuyderland Medical Center^7^, Heerlen, The Netherlands; Duke-NUS Medical School, SINGAPORE

## Abstract

**Background:**

Normal high sensitivity cardiac troponin (hs-cTn) assays rule out acute myocardial infarction (AMI) with great accuracy, but additional non-invasive testing is frequently ordered. This observational study evaluates whether clinical characteristics can contribute to risk stratification and could guide referral for additional testing.

**Methods:**

918 serial patients with acute chest pain and normal hs-cTnT levels were prospectively included. Major adverse cardiac events (MACE) and non-invasive test results were assessed during one-year follow-up. Patients were classified as low and high risk based on clinical characteristics.

**Results:**

MACE occurred in 6.1% of patients and mainly comprised revascularizations (86%). A recent abnormal stress test, suspicious history, a positive family history and higher baseline hs-cTnT levels were independent predictors of MACE with odds ratios of 16.00 (95%CI:6.25–40.96), 16.43 (6.36–42.45), 2.33 (1.22–4.42) and 1.10 (1.01–1.21), respectively. Absence of both recent abnormal stress test and suspicious history identified 86% of patients. These patients were at very low risk for MACE (0.4% in 30-days and 2.3% in one-year). Despite this, the majority (287/345 = 83%) of additional tests were performed in low risk patients, with <10% abnormal test findings. The diagnostic yield was significantly higher in the remaining higher risk patients, 40% abnormal test findings and a positive predictive value of 70% for MACE. Similar results were observed in patients without known coronary artery disease.

**Conclusions:**

Clinical characteristics can be used to identify low risk patients with acute chest pain and normal hs-cTnT levels. Current strategies in the emergency department result in numerous additional tests, which are mostly ordered in patients at very low risk and have a low diagnostic yield.

## Introduction

Acute chest pain is one of the most common reasons for emergency department (ED) visits and is a symptom of a wide variety of diseases, ranging from a trivial ailment to life-threatening disorders. Despite advances in risk stratification, differentiating acute myocardial infarction (AMI) from non-cardiac causes remains challenging. Although only a minority of patients is ultimately diagnosed with AMI, excluding AMI is crucial for appropriate treatment and reducing mortality.[1–6]

The initial evaluation of patients with chest pain is based on clinical history taking, a physical examination and an electrocardiogram (ECG). Although generally regarded as subjective, a combination of these elements may help identifying patients at higher risk.[7] However, sensitivity of the initial evaluation is insufficient to reliably detect all patients with AMI and therefore cardiac biomarker testing (cardiac troponin) is often decisive in the diagnostic approach of patients with acute chest pain.[1,3]

High sensitivity cardiac troponin (hs-cTn) assays allow ruling out of AMI at presentation with high confidence (negative predictive value 99%) and normal hs-cTn levels indicate an excellent prognosis.[8–11] Despite this and endorsed by current guidelines, patients with normal troponin levels are frequently referred for additional non-invasive testing to further reduce the risk of (acute and/or subsequent) AMI and for reassurance of both treating physician and patient.[1,3,12] However, this approach has been questioned, as it is associated with a low diagnostic yield, many false positive test results, higher healthcare costs, increased radiation exposure and no clear benefit in clinical outcome.[13,14]

We hypothesize that commonly available clinical characteristics can reliably risk stratify patients with acute chest pain and normal hs-cTnT levels. Secondly, routine use of non-invasive testing may not be useful in all patients, for the detection of unstable angina, predicting future cardiac events and need for coronary revascularization.

## Marterial and methods

### Study population and objectives

Consecutive patients presenting with acute chest pain at the ED of the Maastricht University Medical Center (MUMC) were prospectively enrolled from April 11, 2012 to April 10, 2013 in this observational cohort trial. The MUMC is a university hospital with a catchment area of 250.000 inhabitants in the South-eastern part of the Netherlands. Although the MUMC is a university hospital, it serves as the sole hospital for patients within the municipality of Maastricht and several surrounding municipalities. Therefore, over 95% of patients are local residents. During the study period, the MUMC was the only hospital with a primary percutaneous coronary intervention (PCI) service in the South-eastern part of the Netherlands and may therefore explain the high rate of patient with ST-elevation MI.

Patients were included if they presented with acute chest pain suggestive of cardiac ischemia and had normal hs-cTnT levels (≤14 ng/L, 5^th^ generation troponin assay, Roche Diagnostics, Basel, Switzerland) at presentation and, if re-testing was clinically indicated, 3 hours after presentation.[1,3] Patients with ST-elevation MI were excluded. All patients in this study received standard care in the work-up for suspected AMI, including a thorough medical history, physical examination, 12-lead ECG, biomarker testing and additional cardiac testing when indicated. This work-up was left at discretion of the attending cardiologist, and based on institutional and European guidelines. The study was approved by the Ethical Committee of the Maastricht University Hospital and Maastricht University (METC identifier 14-4-009). The study was performed and reported in compliance with the STROBE guidelines.

The primary study objective was to identify clinical characteristics that allowed stratification of patients to low and high risk for 30-day and one-year major adverse cardiac events (MACE). Secondly, the result and outcome of additional cardiac tests on admission and during one-year follow-up were evaluated and compared between low and high risk patients.

### Outcome data and definitions

The number of cardiac deaths and spontaneous AMI during 30-day and one-year follow-up were scored as hard cardiac events. MACE was defined as a composite of cardiac death, AMI, previously unplanned revascularization (PCI or CABG) and admission for congestive heart failure during 30-day and one-year follow-up. Cardiac death was a combined endpoint of cardiac deaths and unknown deaths, thus all deaths were considered as being cardiac in origin, unless a clear non-cardiac cause of death was present. AMI was defined as AMI type 1 based on the Universal Definition of AMI, thus related to atherosclerotic plaque rupture resulting in decreased blood flow and myocardial necrosis.[15] Coronary revascularizations planned before index presentation were not counted as an event (n = 12). Congestive heart failure was scored when patients were admitted and treated for decompensated heart failure. In case of multiple events, the first event that occurred in an individual patient was considered as the patient outcome and time to event.

The number of non-invasive tests during index presentation and during the one-year follow-up period were recorded. Tests performed after a MACE occurred were not considered. For the purpose of the current study, a positive test result was defined as 1) a reversible perfusion defect on single-photon emission tomography (SPECT [summed difference score >4%]), 2) ≥1mm ST-segment depression on an electrocardiographic exercise test (EET), 3) extensive coronary calcifications with an Agatston score ≥1000 on CT, 4) a (new) luminal narrowing of ≥70% on cardiac computed tomography angiography (CCTA) and/or invasive coronary angiography (ICA) or abnormal fractional flow reserve (≤0.80)[16]. As no stress echocardiography was being performed during the study period, echocardiograms were not counted as positive or negative for ischemic coronary artery disease (CAD). A test was considered non-diagnostic if the images were uninterpretable or in case of a negative EET at submaximal exercise (<85% of the maximum predicted target heart rate).

### Data analysis and patient chart review

Admission and follow-up data were retrieved from the electronic patient record system by two reviewers. The general practitioner was contacted in case additional information was necessary. A detailed assessment of the medical history including traditional risk factors for CAD, interpretation of the ECG, cardiac biomarkers, additional testing on admission and during follow-up (e.g. EET, echocardiography, CCTA, SPECT, ICA), final diagnosis at discharge from the ED or patient ward, patient treatment and adverse cardiac events were obtained. An independent cardiologist was available to resolve any discrepancies.

The history taking was classified as follows and based on the traditional clinical classification of chest pain:[17] 1) Highly suspicious: typical angina (i.e. substernal chest discomfort, exacerbation of symptoms by physical exertion or stress, relief of symptoms by rest or nitrates) and/or chest pain that was recognized from prior myocardial ischemic episodes, 2) moderately suspicious: atypical angina meeting two of three criteria for typical angina, and 3) slightly suspicious: non-anginal chest pain that meets maximally only one of the criteria for typical angina. This categorization of patients was based on the documentation by the attending cardiologist and review of the patient records.

A history of coronary disease was defined as known coronary artery disease (≥50% stenosis), previous MI or coronary revascularization (PCI or CABG). Traditional risk factors for CAD were scored: 1) hypertension, 2) hypercholesterolemia, 3) diabetes, 4) smoking and a 5) family history of CAD at age ≤65 years old. A stress test (e.g. EET or SPECT) was considered recent if the stress test was performed within 3 months before ED presentation. The diagnosis at discharge (e.g. acute MI, unstable angina, arrhythmia, no acute cardiac pathology) was based on the documentation by the attending cardiologist. The diagnosis “unknown but no acute cardiac pathology” was a combination of the following: 1) acute myocardial injury was excluded, 2) gastro-oesophageal symptoms, 3) musculoskeletal complaints, 4) pleuritis and pneumonia, or 5) hyperventilation syndrome.

Scoring of ECG parameters was based on the interpretation of the ECG by the attending cardiologist at the time of presentation. Cardiac biomarker testing was performed on clinical indication and troponin results at presentation were considered as continuous and commonly accepted dichotomous values: undetectable hs-cTnT level (<5 ng/L) and detectable hs-cTnT levels (5–14 ng/L).

### Statistical analyses

Statistical analysis was performed with SPSS statistics 23 (SPSS Inc., Chicago, Illinois). Categorical data are expressed as frequencies and percentages. Differences in categorical data were evaluated using a Chi-square or in cases where the expected cell count was <5 a Fisher’s Exact Test was used. Continuous variables with normally distributed data are expressed as mean ± SD and differences between groups were compared using an independent *t*-test or one-way ANOVA when more than two groups were compared. Non-normally distributed continuous data were presented as a median with interquartile range (IQR) and differences between groups were tested with the Mann-Whitney U test or independent samples Kruskal-Wallis test when more than two groups were compared. A two-tailed *p*-value of <0.05 was considered statistically significant.

Univariable binary logistic regression was performed to explore the effect of patient characteristics at baseline on the occurrence of MACE during 1-year follow-up. Each characteristic was tested independently in a univariable logistic regression model. For continuous data, odds ratios were expressed per unit of the determinant. Independent variables that were significantly associated with MACE during follow-up were included in the multivariable analysis. Missing data was excluded from the analysis. A variable was selected for a stepwise backward multivariate binary logistic regression based on: 1) a univariable *p*-value <0.01, 2) a favourable univariable odds ratio, and 3) clinical relevance. Variables associated with a p-value <0.10 in the multivariable binary logistic regression model were retained in the model. For variables independently associated with MACE, the cumulative event rate was estimated by a Kaplan-Meier analysis and a log-rank test was used to test for differences between groups.

Patients were classified as low and high risk based on clinical characteristics independently associated with MACE. The number and outcome of additional non-invasive testing was scored and compared between low and high risk patients. A subgroup analysis was performed in patients without known coronary disease.

## Results

**Fig 1:**
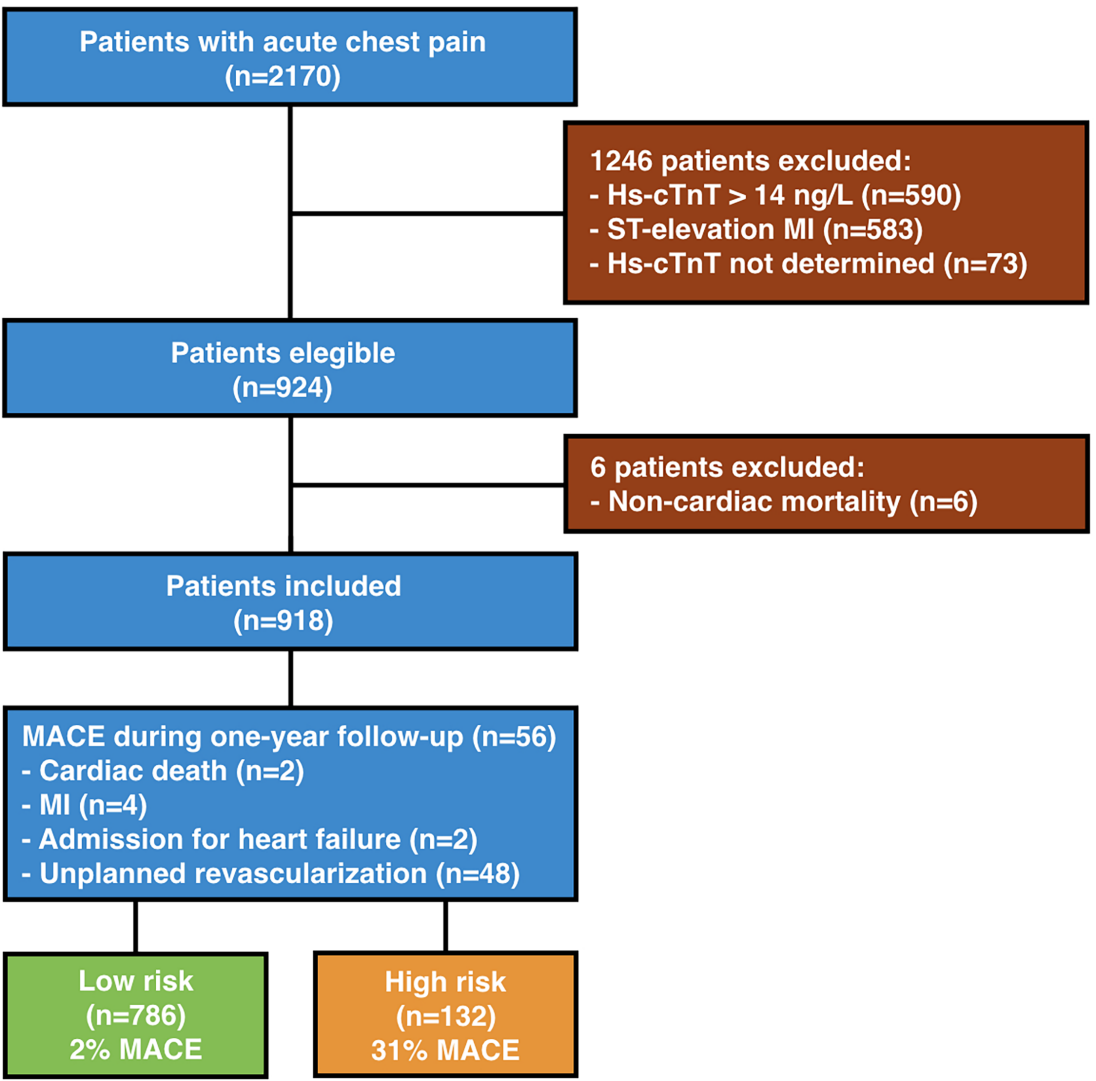
Patient selection flow chart. Hs-cTnT = high sensitivity cardiac troponin T; MI = myocardial infarction.

In total, 2170 patients with acute chest pain suspected for myocardial ischemia were evaluated on the ED during the one-year inclusion period. Patients with ST-elevation AMI (n = 583) or patients with elevated hs-cTnT concentration (n = 590) were excluded from the analysis. An additional 73 patients were excluded because no hs-cTnT measurement was performed. During one-year follow-up, 6 patients died of non-cardiac causes (4 cancer, 1 cerebral haemorrhage, 1 ruptured abdominal aortic aneurysm) and were excluded. The final study population consisted of 918 patients (**Fig 1**). Baseline characteristics of the study population are shown in **Table 1**. Mean age was 59 years and 50% were men. A total of 291 patients (32%) had known coronary disease. Mean hs-cTnT level was 6.9 ± 3.6 ng/L (median 7.0 ng/L [IQR 5–10]). The majority of patients did not have ST-deviations or T-wave inversions on the ECG. A total of 832 patients (91%) were immediately discharged from the ED and had in general a lower clinical risk profile (e.g. atypical history, less frequent history of cardiovascular disease and ECG changes, and lower hs-cTnT levels). No patient was admitted or discharged with a diagnosis of AMI during the initial admission. The most common diagnosis at discharge (88%) was no acute cardiac pathology (**S1 Table**).

**Table 1:** Baseline patient characteristics and univariable predictors for MACE.

Characteristic	All Patients (n = 918)	MACE (n = 59)	No MACE (n = 859)	*P*-value^#^	Univariable OR (95% CI)
**General**					
Age (years)	59.1 ± 12.8	63.2 ± 9.4	58.8 ± 13.0	**0.012**	1.03 (1.01–1.05)
Male gender	454 (49.5%)	35 (59.3%)	419 (48.8%)	0.117	1.53 (0.90–2.62)
BMI	27.9 ± 5.2	27.6 ± 4.7	27.9 ± 5.3	0.691	0.99 (0.93–1.04)
**Clinical history**					
*Known cardiovascular disease*					
- History of revascularization	238 (25.9%)	30 (50.8%)	208 (24.2%)	**<0.001**	3.24 (1.90–5.52)
- History of MI	153 (16.7%)	13 (22.0%)	140 (16.3%)	0.253	1.45 (0.76–2.76)
Risk factors for CAD*					
- Hypertension	414 (45.1%)	33 (55.9%)	381 (44.4%)	0.115	1.53 (0.90–2.60)
- Diabetes mellitus	124 (13.5%)	17 (28.8%)	107 (12.5%)	**0.001**	2.77 (1.52–5.05)
- Hypercholesterolemia	330 (35.9%)	34 (57.6%)	296 (34.5%)	**0.001**	2.27 (1.33–3.87)
- Positive family history	349 (38.0%)	34 (57.6%)	315 (36.7%)	**0.002**	2.59 (1.49–4.51)
- Smoking	412 (44.9%)	28 (47.5%)	384 (44.7%)	0.795	1.07 (0.63–1.82)
**Emergency department presentation**					
Patient history classification				**<0.001**	
- Slightly suspicious	482 (52.5%)	6 (10.2%)	476 (55.4%)		-
- Moderately suspicious	318 (34.6%)	18 (30.5%)	300 (34.9%)		4.76 (1.87–12.13)
- Highly suspicious	118 (12.9%)	35 (59.3%)	83 (9.7%)		33.45 (13.64–82.03)
Recent abnormal stress test	30 (3.3%)	18 (30.5%)	12 (1.4%)	**<0.001**	30.99 (14.00–68.61)
**Electrocardiogram**					
ST-T segment changes	85 (9.3%)	8 (13.6%)	77 (9.0%)	0.239	1.59 (0.73–3.48)
Negative T-wave	102 (11.1%)	13 (22.0%)	89 (10.4%)	**0.006**	2.45 (1.27–4.70)
Normal ECG	685 (74.6%)	40 (67.8%)	645 (75.1%)	0.213	0.70 (0.40–1.23)
**Laboratory Testing**					
Hs-cTnT at baseline (ng/L)	6.9 ± 3.6	8.6 ± 3.0	6.8 ± 3.6	**<0.001**	1.16 (1.07–1.26)
Undetectable hs-cTnT at baseline	205 (22.3%)	5 (8.5%)	200 (23.3%)	**0.008**	0.31 (0.12–0.77)
Delta hs-cTnT (ng/L)^	1.0 (0.0–1.0)	1.0 (0.0–1.3)	1.0 (0.0–1.0)	0.237	1.01 (0.74–1.38)
CK (U/L)	87 (64–120)	79 (68–114)	88 (64–120)	0.610	1.00 (0.99–1.00)
Creatinine (mol/L)	78.0 ± 21.4	74.1 ± 15.3	78.3 ± 21.8	0.265	0.99 (0.97–1.01)

Continuous data are expressed as mean ± standard deviation or median (interquartile range) and given odds ratios are expressed per unit of the determinant. Categorical data are expressed as frequencies with (percentages).

^#^ *P*-values are shown for the comparison of patients without a MACE and patients experiencing a MACE during 1-year follow-up. Significance was calculated by Chi-square test, Fisher’s Exact test, independent *t*-test or Mann-Whitney U test when appropriate.

^ A second hs-cTnT measurement after 3 hours to calculate the change in hs-cTnT level was available in 178 (19%) patients.

*Data on cardiovascular risk factors were missing for 19 patients.

BMI = body mass index; CAD = coronary artery disease; CI = confidence interval; CK = creatine kinase; ECG = electrocardiogram; hs-cTnT = high sensitivity cardiac Troponin-T; MACE = major adverse cardiac events; MI = myocardial infarction; ng/L = nanograms per liter; OR = odds ratio; μmol/L = micromol per liter; U/L = units per liters.

A total of 8 patients experienced cardiac death or AMI (2 unknown deaths and 6 AMI, median time to event was 167 days [IQR 50–302]) resulting in a one-year hard cardiac event rate of 0.9%. One event occurred within the first month after presentation of acute chest pain (death of unknown cause, 6 days after index presentation), resulting in a 30-day hard cardiac event-free survival of 99.9%. MACE occurred in 56 patients (6.1%) during one-year follow-up: 2 cardiac deaths (unknown cause for both), 4 AMI, 2 admissions for heart failure and 48 unplanned revascularizations. The 30-day MACE-free survival was 97.8%, 1 cardiac death and 19 revascularizations.

Notably, undetectable hs-cTnT levels (i.e. <5 ng/L) were observed in 205 patients (22% of the population). Patients with undetectable hs-cTnT levels had an excellent prognosis with a cardiac death and AMI-free survival of 100% and MACE free survival of 97.6% during one-year follow-up.

### Clinical predictors of MACE and risk stratification

**Fig 2:**
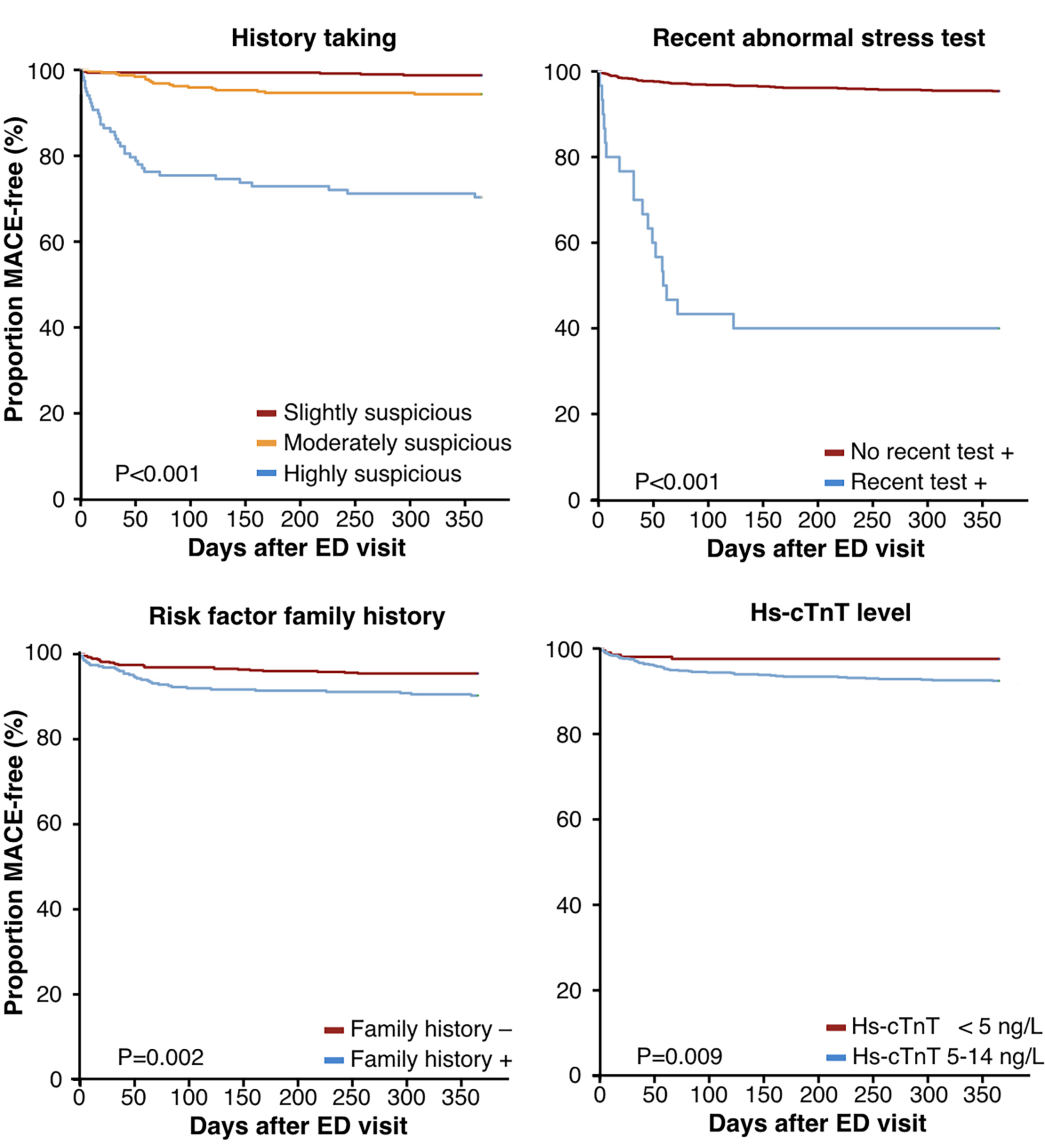
Kaplan-Meier survival curves for variables that were independently associated with MACE-free survival. ED = emergency department; hs-cTnT = high sensitivity cardiac troponin T; MACE = major adverse cardiac events. Note, for clarification of the image is chosen for hs-cTnT detectable vs. undetectable instead of showing the continuous characteristics.

Patients with MACE during follow-up were older, had more frequently a history of coronary revascularization, diabetes mellitus, hypercholesterolemia, family history, suspicious patient history, recent abnormal stress test and higher hs-cTnT levels than patients without MACE (**Table 1**). Multivariable logistic regression analysis demonstrated that a recent abnormal stress test, a more suspicious history, a positive family history and higher hs-cTnT concentrations were independently associated with MACE (**Table 2** and **Fig 2)**.

**Table 2:** Multivariable logistic regression analysis of characteristics associated with the prospective occurrence of MACE.

Variable	Multivariable OR (95% CI)	*P*-value
Recent abnormal stress test	16.00 (6.25–40.96)	<0.001
Highly suspicious history	16.43 (6.36–42.45)	<0.001
Moderately suspicious history	2.89 (1.09–7.63)	0.033
Positive family history	2.32 (1.22–4.42)	0.010
Hs-cTnT level	1.10 (1.01–1.21)	0.034
Risk factor diabetes mellitus	-	-
Risk factor hypercholesterolemia	-	-
History of revascularization	-	-
Negative T-wave on ECG	-	-

CI = confidence interval; hs-cTnT = high sensitivity cardiac Troponin-T; MACE = major adverse cardiac events; ng/L = nanograms per liter; OR = odds ratio. Risk factor diabetes mellitus, negative T-wave on the ECG, risk factor hypercholesterolemia and history of revascularization were eliminated in a backward multivariable logistic regression analysis as it did not significantly contribute to the model.

A recent abnormal stress test and highly suspicious history independently predicted MACE with high odds ratios of 16.00 (6.25–40.96) and 16.43 (6.36–42.45), respectively. A simple clinical rule based on the absence (low risk) or presence of at least one of these two variables (high risk) allowed us to risk stratify patients. The vast majority of patients (86%) did not have a recent abnormal stress test or highly suspicious history and were classified as low risk. These patients had an overall 30-day MACE rate of 0.4% and a one-year MACE rate of 2.3% (1.7% revascularizations). The remaining patients (14%) were classified as high risk, having a one-year MACE rate of 31.1% (28.8% revascularizations). Moreover, the one-year event rate for cardiac death and AMI was significantly different between very low and higher risk patients (0.5% vs. 3.0%, p = 0.018).

### Frequency and usefulness of additional non-invasive testing

Over one third of patients underwent at least one non-invasive diagnostic test to detect ischemia or CAD (**Table 3A**). EET was performed in 30% of patients and comprised the largest proportion of all non-invasive tests applied. The number of patients undergoing additional testing increased to 51% if echocardiography was included as well. Over one third of these patients underwent more than one non-invasive test. Patients with at least one non-invasive diagnostic test had rather similar baseline characteristics in comparison with those without additional testing, except for mild variations in the number of patients with hypercholesterolemia (32.1% vs. 42.3%, p = 0.003), a positive family history (35.3% vs. 42.6%, p = 0.039) and an atypical history (45.8% vs. 56.5%, p = 0.007, **S2 Table**). The proportion of patients with cardiac death and AMI during follow-up did not significantly differ between patients with and without additional non-invasive testing (0.9% vs. 0.9%, p = 0.996). Referral to a specific non-invasive test mostly depended on the presence of known cardiovascular disease. Patients referred to SPECT tend to have a higher clinical risk profile than patients referred for EET and CCTA (**S3 Table**).

**Table 3:** Additional tests during index presentation and one-year follow-up in all patients (A) and stratified for risk profile as identified by the new simple clinical rule based on the absence of a recent abnormal stress test and highly suspicious history (low risk) or presence of at least one of these characteristics (B).

**A**	**Total population (n = 918–59 MACE)**					
**Test**	**Number of tests** **(%)**	**Abnormal tests** **(%)**	**PPV MACE** **(%)**			
**EET**	271 (30%)	17 (6%)	59%			
**CCTA**	86 (9%)	17 (20%)	41%			
**SPECT**	45 (5%)	13 (29%)	23%			
**≥1 CCTA, EET or SPECT**	345 (38%)	46 (13%)	43%			
**Echo**	251 (27%)					
**≥1 CCTA, EET, SPECT or Echo**	465 (51%)					
**Primary ICA**	91 (10%)	34 (37%)	68%			
**≥1 CCTA, EET, SPECT or primary ICA**	431 (47%)	78 (18%)	54%			
**B**	**Low risk (n = 786–18 MACE)**			**High risk (n = 132–41 MACE)**		
**Test**	**Number of tests** **(%)**	**Abnormal tests** **(%)**	**PPV MACE** **(%)**	**Number of tests** **(%)**	**Abnormal tests** **(%)**	**PPV MACE** **(%)**
**EET**	222 (28%)	4 (2%)	0%	49 (37%)	13 (10%)	77%
**CCTA**	78 (10%)	12 (15%)	25%	8 (7%)	5 (63%)	80%
**SPECT**	33 (4%)	8 (24%)	13%	12 (9%)	5 (42%)	40%
**≥1 CCTA, EET or SPECT**	287 (37%)	23 (8%)	17%	58 (44%)	23 (40%)	70%
**Echo**	207 (26%)			44 (33%)		
**≥1 CCTA, EET, SPECT or Echo**	385 (49%)			80 (61%)		
**Primary ICA**	39 (5%)	4 (10%)	25%	52 (39%)	30 (58%)	73%
**≥1 CCTA, EET, SPECT or primary ICA**	326 (41%)	27 (8%)	19%	105 (80%)	51 (49%)	73%

Categorical data are expressed as frequencies with (percentages). CCTA = computed tomography angiography; Echo = echocardiography; EET = electrocardiographic exercise test; ICA = invasive coronary angiography; MACE = major adverse cardiac events; SPECT = single-photon emission computed tomography. Primary ICA means immediate referral to ICA without performing non-invasive cardiac imaging first. ≥1 CCTA, EET or SPECT means: at least one CCTA, EET or SPECT scan was performed in a single patient.

The majority of tests (83%) were performed in patients identified as low risk by our simple clinical rule (**Table 3B**). A severe coronary stenosis or detection of myocardial ischemia (by CCTA or EET/SPECT, respectively) was found in 8% of low risk patients and in 40% of high risk patients, p<0.001. The positive predictive value of non-invasive testing to predict MACE was 17% for patients classified as low risk and 70% for patients at high risk, p<0.001. Patients in the high-risk category were more frequently referred for ICA and the proportion of patients with obstructive CAD was higher (10% versus 58%, respectively, p<0.001).

In low risk patients (n = 786), suspicion of obstructive CAD on non-invasive testing (n = 23) was confirmed by ICA in 6 patients of whom 4 underwent revascularization. Furthermore, anti-platelet therapy was initiated or intensified in 10 patients. Another 39 patients were directly referred to ICA (i.e. without non-invasive testing first) and obstructive CAD was found in 4 patients. One of these 4 was revascularized, which was complicated by an ST-elevation MI due to an in-stent thrombus two weeks after the intervention. In summary, 326 tests in 786 low risk patients led to 5 revascularization procedures and 6 other patients received optimized medical therapy (3% of low risk patients undergoing testing). The characteristics and test details of all patients at low risk with a positive non-invasive or primary invasive test result are presented in **S4 Table**.

### Observations in patients without known coronary disease

In total 627 patients did not have a history of myocardial infarction, revascularization or obstructive CAD. One-year MACE-rate was significantly lower in these patients (n = 26 [4.1%] of whom 3 experienced cardiac death or acute MI) compared with patients with a history of coronary disease (n = 33 [11.3%], p<0.001). Undetectable troponin levels implied an excellent prognosis as 1 out of 161 experienced MACE (0.6%). Multivariable logistic regression analysis demonstrated that a recent abnormal stress test, a more suspicious history, a positive family history, a negative T wave and higher hs-cTnT concentrations were independently associated with MACE. The simple clinical rule identified 591 patients (94%) as low risk having a 30-day MACE rate of 0.3% and a one-year MACE rate of 1.7% (1.4% revascularizations). High risk patients had a one-year MACE rate of 44.4%.

Patients with traditional cardiovascular risk factors present tended to undergo non-invasive diagnostic testing more frequently. Of 226 patients (36%) referred for additional testing, 211 patients (93%) were performed in patients classified as low risk and 26 of 226 tests (12%) were abnormal. The diagnostic yield was considerably higher in patients classified as high risk (67% abnormal test results) versus patients classified as low risk (8% abnormal test results, p<0.001).

## Discussion

This study identifies low and high risk patients based on clinical characteristics in a real-world acute chest pain population with normal hs-cTnT levels and evaluates the value of additional testing in relation to patient outcome. The vast majority of patients can be classified as low risk (86%) with a 30-day MACE rate of 0.4%. Despite this very low risk of events, additional testing was commonly performed. Low risk patients are unlikely to benefit from additional testing, as abnormal test findings and therapeutic interventions are infrequent and the predictive value for MACE is low. Our results open the discussion for restricting downstream testing in patients with normal hs-cTnT levels, especially in those identified as low risk.

The incremental value of non-invasive cardiac testing in acute chest pain with normal cardiac troponins has been questioned.[13,14] Concerns on routine use of non-invasive imaging are fuelled by the lack of improvement in patient outcome, increased ionizing radiation exposure, increase in potential harmful downstream invasive procedures and higher health care costs.[18]

Our results confirm the excellent negative predictive value of hs-cTnT assays to rule out acute MI, as none of the patients was initially admitted or discharged with an AMI.[8–11] However, we extend prior research with the finding of adverse events (including revascularizations) in 6% of the population during one-year follow-up. Risk scores may aid in identifying those patients with adverse outcome. An overwhelming number of risk scores and pathways have been proposed for risk stratification over the last years, such as GRACE, TIMI, ADAPT, HEART, No Objective Testing rule, ESC 0h/1h, High-STEACS.[3,19–27] Similar to our clinical rule are most of these scores highly accurate in identifying patients at low risk for adverse outcome. Different from these scores, our clinical rule requires only 2 variables (i.e. recent abnormal stress test result and highly suspicious clinical history) that are either present (high risk) or absent (low risk if both are absent).

Despite that some studies suggest that additional testing may not be necessary in patients classified as low risk, such as the No Objective Testing rule, only very little data is available on the diagnostic yield of additional testing in patients identified as low risk.[23] One of the few available studies is by Mahler et al. investigating the HEART Pathway, which recommended discharge without additional testing in patients with a low HEART score and negative serial troponin levels. The HEART Pathway resulted in a reduction of additional testing (minus 12%), but was not powered to test differences in MACE.[28] The proposed simple clinical rule in the current manuscript identifies 86% of patients at such a low risk that additional testing may not be necessary, reducing the number of additional tests substantially.

The current study uniquely combines the role of a clinical rule in risk stratification of patients with acute chest pain and normal high-sensitivity troponin levels and the diagnostic yield of additional testing based on this risk stratification. Others have shown that additional testing does not improve outcome in low risk chest pain patients.[29,30] In a large health insurance claim data cohort additional testing was associated with an increased number of invasive coronary angiography and revascularization, while number of MI remained similar.[30] These findings were confirmed in a retrospective study in almost 1 million patients.[31] This suggests that overdiagnosing may trigger overtreatment with no benefit on patient outcome. Another large observational study in over 200 hospitals showed that hospitals with higher rates of additional cardiac imaging, did not have fewer readmissions for AMI.[32] In addition, systematically using CCTA in patients with low risk acute chest pain did not improve patient outcome, but resulted in more invasive procedures and higher costs.[33–36] A large multi-centre controlled trial confirming all these observational and retrospective findings could fill the unmet clinical need for effective referral for downstream testing.

The rational of performing additional testing in patients with acute chest pain is to detect obstructive CAD as early as possible, which may trigger adequate treatment and hereby reduce the number of acute MI and cardiac death. However, as described above there is no evidence that applying routine non-invasive testing improves prognosis, although arguably a higher risk subgroup might benefit from this approach. Besides no evidence for improvement in prognosis, the diagnostic yield of routine additional cardiac imaging is generally low and many false positive test results are encountered.[37–40] The current study shows the diagnostic yield of additional testing could improve when selecting high risk patients. However, one should keep in mind that even in case obstructive CAD is found, it is not yet clear whether this should be treated with medication alone or in combination with coronary revascularization.[41]

Some limitations of the current study should be considered. Firstly, this is a single centre observational study in an unselected population with acute chest pain and clinical decisions were made irrespective of our findings. The description of real world data has important merits in terms of generalizability of results. But no patient was discharged or tested based on our findings, thus the efficacy of our protocol remains unknown. Our hypothesis that low risk patients may be discharged without further testing requires further investigation preferably in a multicentre randomized clinical setting using multiple hs-cTn assays. Secondly, patients who were not hospitalized were not routinely scheduled for re-evaluation. Therefore potentially, although unlikely, an AMI may have been missed. Thirdly, a number of revascularizations might be driven by patient symptoms and an anatomical stenosis, not by evidence of myocardial ischemia. In addition, progression of known CAD (i.e. rather stable CAD) may have triggered ED presentation or even revascularization. To exclude confounding of known CAD, a separate subgroup analysis in patients without known CAD was performed. The results regarding variables independently predicting MACE, risk stratification using the simple clinical rule and additional diagnostic testing and test results were relatively analogous to the unselected acute chest pain population including patients with a known history of CAD.

## Conclusions

Patients with acute chest pain and normal hs-cTnT levels have an excellent prognosis. Based on clinical characteristics can majority of patients be considered as extremely low risk. In such low risk patients, has additional testing a very limited yield. Based on clinical characteristics, a smaller subset of patients may be considered as higher risk for MACE with potential benefit of additional testing. This clear-cut and easy to implant approach could significantly reduce the number of often unnecessary additional tests, potentially reduce the number of hospital admissions and result in a more cost-effective approach in these patients.

## Supporting information

S1 Table Diagnosis at discharge from the emergency department. (DOCX)

S2 Table Baseline patient characteristics for scheduling additional testing. (DOCX)

S3 Table Baseline patient characteristics stratified for each non-invasive test performed. (DOCX)

S4 Table Patient characteristics of patient identified as low risk with an abnormal EET, CCTA, SPECT or primary ICA (n = 27). (DOCX)

S1 Database Data supporting main study findings. (XLSX)

## Abbreviations

AMI: acute myocardial infarction
CABG: coronary artery bypass grafting
CAD: coronary artery disease
CCTA: cardiac computed tomography angiography
ECG: electrocardiogram
ED: emergency department
EET: electrocardiographic exercise testing
Hs-cTn: high sensitivity cardiac troponin
ICA: invasive coronary angiography
MACE: major adverse cardiac events
PCI: percutaneous coronary intervention
SPECT: single-photon emission computed tomography
