# Supplementary material for: Risk stratification and role for additional diagnostic testing in patients with acute chest pain and normal high-sensitivity cardiac troponin levels
Source: PLoS One. 2018 Sep 7;13(9):e0203506. doi: 10.1371/journal.pone.0203506 (PMC6128560; doi:10.1371/journal.pone.0203506)
Supplement: S1 Table — (DOCX) [file pone.0203506.s001.docx]

**S1 Table.** Diagnosis at discharge from
the emergency department.

| **Diagnosis at discharge** |  |
| --- | --- |
| Unknown but no acute cardiac pathology | 87.8% |
| Unstable angina pectoris | 5.7% |
| Pericarditis | 1.9% |
| Arrhythmia | 1.8% |
| Hypertension | 1.7% |
| Variant angina | 0.4% |
| Other | 0.5% |

| The final diagnosis in patients with “no acute cardiac pathology” includes gastro-esophageal disease and musculoskeletal pathology. |
| --- |
