# Supplementary material for: Risk stratification and role for additional diagnostic testing in patients with acute chest pain and normal high-sensitivity cardiac troponin levels
Source: PLoS One. 2018 Sep 7;13(9):e0203506. doi: 10.1371/journal.pone.0203506 (PMC6128560; doi:10.1371/journal.pone.0203506)
Supplement: S2 Table — (DOCX) [file pone.0203506.s002.docx]

**S2 Table**. Baseline patient characteristics for scheduling additional testing.

| **Characteristic** | **All Patients  (n=918)** | **Patients without a test (n=573)** | **Patients with ≥1 EET, CCTA or SPECT (n=345)** | ***P*-value^#^** |
| --- | --- | --- | --- | --- |
| **General** |  |  |  |  |
| Age (years) | 59.1 ± 12.8 | 59.2 ± 13.9 | 59.0 ± 10.8 | 0.875 |
| Male gender | 454 (49.5%) | 278 (48.5%) | 176 (51.0%) | 0.463 |
| BMI | 27.9 ± 5.2 | 27.8 ± 5.4 | 28.0 ± 5.0 | 0.732 |
| **Clinical history** |  |  |  |  |
| *Known cardiovascular disease* |  |  |  |  |
| - History of revascularization | 238 (25.9%) | 140 (24.4%) | 98 (28.4%) | 0.183 |
| - History of MI | 153 (16.7%) | 95 (16.6%) | 58 (16.8%) | 0.927 |
| *Risk factors for CAD** |  |  |  |  |
| - Hypertension | 414 (45.1%) | 245 (42.8%) | 169 (49.0%) | 0.099 |
| - Diabetes | 124 (13.5%) | 70 (12.2%) | 54 (15.7%) | 0.165 |
| - Hypercholesterolemia | 330 (35.9%) | 184 (32.1%) | 146 (42.3%) | **0.003** |
| - Positive family history | 349 (38.0%) | 202 (35.3%) | 147 (42.6%) | **0.039** |
| - Smoking | 412 (44.9%) | 259 (45.2%) | 153 (44.3%) | 0.651 |
| **Emergency department presentation** |  |  |  |  |
| Patient history classification |  |  |  | **0.007** |
| - Slightly suspicious | 482 (52.5%) | 324 (56.5%) | 158 (45.8%) |  |
| - Moderately suspicious | 318 (34.6%) | 183 (31.9%) | 135 (39.1%) |  |
| - Highly suspicious | 118 (12.9%) | 66 (11.5%) | 52 (15.1%) |  |
| Recent abnormal stress test | 30 (3.3%) | 18 (3.1%) | 12 (3.5%) | 0.781 |
| **Electrocardiogram** |  |  |  |  |
| ST-T segment changes | 85 (9.3%) | 57 (9.9%) | 28 (8.1%) | 0.354 |
| Negative T-wave | 102 (11.1%) | 60 (10.5%) | 42 (12.2%) | 0.427 |
| Normal ECG | 685 (74.6%) | 431 (75.2%) | 254 (73.6%) | 0.591 |
| **Laboratory Testing** |  |  |  |  |
| Hs-cTnT at baseline (ng/L) | 6.9 ± 3.6 | 7.0 ± 3.6 | 6.9 ± 3.5 | 0.726 |
| Undetectable hs-cTnT at baseline | 205 (22.3%) | 128 (22.3%) | 77 (22.3%) | 0.994 |
| Delta hs-cTnT (ng/L)^ | 1.0 (0.0-1.0) | 1.0 (0.0-1.0) | 1.0 (0.0-1.0) | 0.157 |
| CK (U/L) | 87 (64-120) | 85.5 (64.0-120.8) | 88.5 (65.0-117.0) | 0.764 |
| Creatinine (μmol/L) | 78.0 ± 21.4 | 78.7 ± 22.6 | 76.7 ± 18.7 | 0.358 |
| **Outcome** |  |  |  |  |
| Cardiac death or MI | 8 (0.9%) | 5 (0.9%) | 3 (0.9%) | 0.996 |
| MACE | 59 (6.4%) | 29 (5.1%) | 30 (8.7%) | **0.030** |

Continuous data are expressed as mean ± standard deviation or median (interquartile range). Categorical data are expressed as frequencies with (percentages).

^#^ *P*-values are shown for the comparison of patients without a MACE and patients experiencing a MACE during 1-year follow-up. Significance was calculated by Chi-square test, Fisher’s Exact test, independent *t*-test or Mann-Whitney U test when appropriate.

^ A second hs-cTnT measurement after 3 hours to calculate the change in hs-cTnT level was available in 178 (19%) patients.

*Data on cardiovascular risk factors were missing for 19 patients.

BMI = body mass index; CAD = coronary artery disease; CCTA = cardiac computed tomography angiography; CI = confidence interval; CK = creatine kinase; ECG = electrocardiogram; EET = electrocardiographic exercise testing; hs-cTnT = high sensitivity cardiac Troponin-T; MACE = major adverse cardiac events; MI = myocardial infarction; ng/L = nanograms per liter; OR = odds ratio; SPECT = single-photon emission computed tomography; μmol/L = micromol per liter; U/L = units per liters.
