# Supplementary material for: Risk stratification and role for additional diagnostic testing in patients with acute chest pain and normal high-sensitivity cardiac troponin levels
Source: PLoS One. 2018 Sep 7;13(9):e0203506. doi: 10.1371/journal.pone.0203506 (PMC6128560; doi:10.1371/journal.pone.0203506)
Supplement: S3 Table — (DOCX) [file pone.0203506.s003.docx]

**S3 Table**. Baseline patient characteristics stratified for each non-invasive test performed.

| **Characteristic** | **EET (n=271)** | **CCTA (n=86)** | **SPECT (n=45)** | ***P*-value overall^#^** |
| --- | --- | --- | --- | --- |
| **General** |  |  |  |  |
| Age (years) | 58.7 ± 10.8 | 57.3 ± 9.4 | 63.1 ± 9.7 | **0.008** |
| Male gender | 153 (56.5%) | 33 (38.4%) | 23 (51.1%) | **0.014** |
| BMI | 27.9 ± 5.1 | 28.0 ± 4.1 | 27.8 ± 5.3 | 0.975 |
| **Clinical history** |  |  |  |  |
| *Known cardiovascular disease* |  |  |  |  |
| - History of revascularization | 82 (30.3%) | 3 (3.5%) | 26 (57.8%) | **<0.001** |
| - History of MI | 49 (18.1%) | 4 (4.7%) | 11 (24.4%) | **0.003** |
| *Risk factors for CAD** |  |  |  |  |
| - Hypertension | 133 (49.1%) | 31 (36.0%) | 28 (62.2) | **0.008** |
| - Diabetes | 42 (15.5%) | 8 (9.3%) | 11 (24.4%) | 0.061 |
| - Hypercholesterolemia | 117 (43.2%) | 26 (30.2%) | 23 (51.1%) | **0.028** |
| - Positive family history | 113 (41.7%) | 46 (53.5%) | 17 (37.8%) | 0.140 |
| - Smoking | 128 (47.2%) | 41 (47.7%) | 15 (33.3%) | 0.224 |
| **Emergency department presentation** |  |  |  |  |
| Patient history classification |  |  |  | 0.078 |
| - Slightly suspicious | 126 (46.5%) | 44 (51.2%) | 16 (35.6%) |  |
| - Moderately suspicious | 101 (37.3%) | 35 (40.7%) | 17 37.8%) |  |
| - Highly suspicious | 44 (16.2%) | 7 (8.1%) | 12 (26.7%) |  |
| Recent abnormal stress test | 10 (3.7%) | 2 (2.3%) | 0 (0.0%) | 0.372 |
| **Electrocardiogram** |  |  |  |  |
| ST-T segment changes | 20 (7.4%) | 4 (4.7%) | 4 (8.9%) | 0.595 |
| Negative T-wave | 32 (11.8%) | 9 (10.5%) | 7 (15.6%) | 0.690 |
| Normal ECG | 200 (73.8%) | 66 (76.7%) | 32 (71.1%) | 0.765 |
| **Laboratory Testing** |  |  |  |  |
| Hs-cTnT at baseline(ng/L) | 6.8 ± 3.4 | 6.4 ± 3.6 | 7.8 ± 3.6 | 0.068 |
| Undetectable hs-cTnT at baseline | 62 (22.9%) | 25 (29.1%) | 7 (15.6%) | 0.209 |
| Delta hs-cTnT (ng/L)^ | 1.0 (0.0-1.0) | 1.0 (0.0-1.0) | 1.0 (0.0-1.0) | 0.579 |
| CK (U/L) | 90 (66-121) | 86 (66-113) | 93 (66-117) | 0.773 |
| Creatinine (μmol/L) | 78.0 ± 18.9 | 70.8 ± 14.7 | 78.0 ± 22.9 | 0.086 |
| **Outcome** |  |  |  |  |
| Cardiac death or MI | 3 (1.1%) | 0 (0.0%) | 1 (2.2%) | 0.452 |
| MACE | 25 (9.2%) | 8 (9.3%) | 5 (11.1%) | 0.922 |

Continuous data are expressed as mean ± standard deviation or median (interquartile range). Categorical data are expressed as frequencies with (percentages). Note that some patients did undergo multiple tests.

^#^ *P*-values are shown for the comparison of patients without a MACE and patients experiencing a MACE during 1-year follow-up. Significance was calculated by Chi-square test, one-way ANOVA test or independent samples Kruskal-Wallis test when appropriate.

^ A second hs-cTnT measurement after 3 hours to calculate the change in hs-cTnT level was available in 82 (20%) patients.

*Data on cardiovascular risk factors were missing for 5 patients.

BMI = body mass index; CAD = coronary artery disease; CCTA = cardiac computed tomography angiography; CI = confidence interval; CK = creatine kinase; ECG = electrocardiogram; EET = electrocardiographic exercise testing; hs-cTnT = high sensitivity cardiac Troponin-T; MACE = major adverse cardiac events; MI = myocardial infarction; ng/L = nanograms per liter; OR = odds ratio; SPECT = single-photon emission computed tomography; μmol/L = micromol per liter; U/L = units per liters.
