# Supplementary material for: Risk stratification and role for additional diagnostic testing in patients with acute chest pain and normal high-sensitivity cardiac troponin levels
Source: PLoS One. 2018 Sep 7;13(9):e0203506. doi: 10.1371/journal.pone.0203506 (PMC6128560; doi:10.1371/journal.pone.0203506)
Supplement: S4 Table — (DOCX) [file pone.0203506.s004.docx]

**S4 Table**. Patient characteristics of patient identified as low risk with an abnormal EET, CCTA, SPECT or primary ICA (n=27).

| **No.** | **Abnormal test** | **Prior medication** | **Other non-invasive tests** | **ICA most severe stenosis** | **Medication change** | | **MACE** | **Comment** |
| --- | --- | --- | --- | --- | --- | --- | --- | --- |
| **Non-invasive cardiac imaging test (n=23)** | | | | | | | | |
| 1 | **EET**  ST-depression | Aspirin, b-blocker, statin | - | LAD mid 50% | - | | No | ICA performed before EET |
| 2 | **EET**  ST-depression | Aspirin, thienopyridine, statin, nitrate, ACEi | SPECT SDS10% | - | - | | No | EET performed before SPECT |
| 3 | **EET**  ST-depression | B-blocker, statin, ACEi, diuretic | Echo | MO1 20-50% | Start: aspirin. Dose change: b-blocker, statin | | No | - |
| 4 | **EET**  ST-depression | Aspirin, b-blocker, ACEi, nitrate, ca-blocker | Echo | RCA prox 50-70% stable CAD | Start: thienopyridine | | No | - |
| 5 | **CCTA**  Agatston ≥1000 | ACEi | EET negative | RPL 50-70% | Start: aspirin, statin, b-blocker, nitrate | | No | EET performed before CCTA |
| 6 | **CCTA**  Agatston ≥1000 | Aspirin, b-blocker, statin, nitrate, dipyridamole | Echo | RCA 100%, LCx 70-90% | Start: thienopyridine. Dose change: nitrate | | No | No revascularization due to severe calcifications |
| 7 | **CCTA**  Agatston ≥1000 | Aspirin, b-blocker, statin, ARB | Echo | - | Dose change: b-blocker, ARB | | No | Asymptomatic follow-up, severe hypertension |
| 8 | **CCTA**  Agatston ≥1000 | Aspirin, statin, ACEi, ca-blocker | - | LAD 50% FFR - | - | | No | Asymptomatic after proton pomp inhibitor |
| 9 | **CCTA**  LCx ≥70% | Aspirin, b-blocker, statin, ARB | EET negative | MO2 70-90% | Start: ARB | | No | EET performed before CCTA |
| 10 | **CCTA**  D1 ≥70% | Aspirin, b-blocker, statin, ARB, ca-blocker, diuretic | EET negative, echo | - | - | | No | EET performed before CCTA |
| 11 | **CCTA**  D1 ≥70% | Aspirin, b-blocker, statin, nitrate, ACEi, ca-blocker | EET non-conclusive, echo | LAD 50-60% | - | | No | EET performed before CCTA |
| 12 | **CCTA**  RCA ≥70% | - | - | - | Start: aspirin, statin | | No | Asymptomatic follow-up |
| 13 | **CCTA**  LAD ≥70% | - | - | LAD 20-50% | Start: aspirin, b-blocker, statin | | No | Follow-up gastroscopy: Barrett’s esophagus |
| 14 | **CCTA**  LAD ≥70% | Aspirin, b-blocker, statin, ACEi, ca-blocker | EET negative | LAD 70-90% | Start: thienopyridine | | Revascularization | - |
| 15 | **CCTA**  RCA ≥70% | B-blocker, statin, ACEi, diuretic, coumarin | EET negative | LAD ≥70% FFR+ AL 70-90% FFR- | Start: thienopyridine | | Revascularization | EET performed before CCTA |
| 16 | **CCTA**  LAD ≥70% | Aspirin, b-blocker, statin, nitrate, ca-blocker | Echo | LAD 70-90% | Start: thienopyridine | | Revascularization | - |
| 17 | **SPECT**  SDS 7% | B-blocker, ARB, nitrate, ca-blocker, coumarin | - | - | - | | No | - |
| 18 | **SPECT**  SDS 12% | Aspirin, statin, ARB, ca-blocker, diuretic | - | RCA 20-50% | Start: b-blocker | | No | - |
| 19 | **SPECT**  SDS 5% | Statin, ACEi | EET non-conclusive, echo | - | Start: aspirin | | No | EET performed before SPECT |
| 20 | **SPECT**  SDS 6% | Aspirin, thienopyridine, b-blocker, statin, ARB, nitrate | EET negative, echo | - | Start: ca-blocker | | No | EET performed before SPECT |
| 21 | **SPECT**  SDS 5% | ACEi, diuretic | Echo | Normal | - | | No | - |
| 22 | **SPECT**  SDS 6% | Aspirin, thienopyridine, b-blocker, statin, nitrate, ca-blocker, coumarin | - | - | - | | No | Asymptomatic follow-up |
| 23 | **SPECT**  SDS 6% | Aspirin, b-blocker | EET negative, echo | RCA 70-90% LCx 70-90% | Start: thienopyridine | | Revascularization | EET performed before SPECT |
|  |  |  |  |  |  | |  |  |
|  |  |  |  |  |  | |  |  |
| **Supplemental Table 4 Continued.** | | | | | | | | |
| **No.** | **Abnormal test** | **Prior medication** | **Other non-invasive tests** | **ICA most severe stenosis** | | **Medication change** | **MACE** | **Comment** |
| **Primary invasive coronary angiography (n=4)** | | | | | | | | |
| 24 | **ICA** | Thienopyridine, b-blocker, ACEi, Statin, nitrate, diuretic, coumarin | Echo | Graft LCx 100% | | - | No | Prior CABG & graft LCx 99% stenosis, progressive to 100% |
| 25 | **ICA** | Aspirin, thienopyridine, b-blocker, statin, nitrate, ca-blocker | Echo | RCA 70-90% | | - | Revascularization | Chronic calcified lesion, after 13 days STEMI: in-stent thrombus |
| 26 | **ICA** | Thienopyridine, b-blocker, statin, ARB, nitrate, coumarin | - | MO1 70-90% | | - | No | ICA was planned before ED visit, MO1 too small for PCI |
| 27 | **ICA** | Aspirin, thienopyridine, b-blocker, statin, ca-blocker, diuretic | Echo | RCA 95% | | Start: ACEi | No | Echo performed after ICA. Asymptomatic follow-up. |

ACEi = angiotensin converting enzyme inhibitor; ARB = angiotensin receptor blockers; b-blokker = beta blocker; ca-blocker = calcium blocker; CAD = coronary artery disease; CCTA = computed tomography angiography; D1 = first diagonal branch; ECG = electrocardiogram; Echo = echocardiography; EET = electrocardiographic exercise test; FFR = fractional flow reserve; ICA = invasive coronary angiography after abnormal non-invasive test result; LAD = left anterior descending; LCx = left circumflex; MACE = major adverse cardiac events; MO1 = first obtuse marginal branch; PCI = percutaneous coronary intervention; RCA = right coronary artery; SDS = summed difference score; SPECT = single-photon emission computed tomography; STEMI = ST-elevation myocardial infarction.
